# Incident Pregnancy and Time to Death or AIDS among HIV-Positive Women Receiving Antiretroviral Therapy

**DOI:** 10.1371/journal.pone.0058117

**Published:** 2013-03-08

**Authors:** Daniel Westreich, Mhairi Maskew, Denise Evans, Cindy Firnhaber, Pappie Majuba, Ian Sanne

**Affiliations:** 1 Department of Obstetrics and Gynecology and Duke Global Health Institute, Duke University, Durham, North Carolina, United States of America; 2 Health Economics and Epidemiology Research Office, Department of Medicine, Faculty of Health Sciences, University of the Witwatersrand, Johannesburg, South Africa; 3 Clinical HIV Research Unit, Department of Medicine, Faculty of Health Sciences, University of the Witwatersrand, Johannesburg, South Africa; 4 Right to Care, Johannesburg, South Africa; University of Cape Town, South Africa

## Abstract

**Background:**

Little is known about the impact of pregnancy on response to highly active antiretroviral therapy (HAART) in sub-Saharan Africa. We examined the effect of incident pregnancy after HAART initiation on clinical response to HAART.

**Methods:**

We evaluated a prospective clinical cohort of adult women initiating HAART in Johannesburg, South Africa between 1 April 2004 and 31 March 2011, and followed up until an event, transfer, drop-out, or administrative end of follow-up on 30 September 2011. Women over age 45 and women who were pregnant at HAART initiation were excluded from the study. Main exposure was having experienced pregnancy after HAART initiation; main outcome was death and (separately) death or new AIDS event. We calculated adjusted hazard ratios (HRs) and 95% confidence limits (CL) using marginal structural Cox proportional hazards models.

**Results:**

The study included 7,534 women, and 20,813 person-years of follow-up; 918 women had at least one recognized pregnancy during follow-up. For death alone, the weighted (adjusted) HR was 0.84 (95% CL 0.44, 1.60). Sensitivity analyses confirmed main results, and results were similar for analysis of death or new AIDS event. Incident pregnancy was associated with a substantially reduced hazard of drop-out (HR = 0.62, 95% CL 0.51, 0.75).

**Conclusions:**

Recognized incident pregnancy after HAART initiation was not associated with increases in hazard of clinical events, but was associated with a decreased hazard of drop-out. High rates of pregnancy after initiation of HAART may point to a need to better integrate family planning services into clinical care for HIV-infected women.

## Introduction

The majority of individuals living with HIV in sub-Saharan Africa are women, most of whom are of reproductive age. [1] In South Africa, where one-sixth of all HIV-infected individuals in the world live, HIV is particularly common among young women and most especially young pregnant women [2], [3] among whom prevalence was estimated at nearly 30% nationally in 2008 [4]. Pregnancy is an indication for HAART initiation [5], and in addition pregnancy is common after HAART initiation [6], [7], [8], [9], [10]. We estimated previously that of women ages 18–25 initiating HAART, 44% would have an incident pregnancy within four years [11], while a recent study by Myer et al. estimated that use of HAART was associated with a 70% higher rate of pregnancy (adjusted hazard ratio 1.7, 95% confidence limits 1.2, 2.5) [8].

While numerous studies have examined optimal methods for prevention of mother to child transmission of HIV and subsequent response to HAART [12], [13], [14], [15], [16], [17], [18] as well as the impact of pregnancy on outcomes of HIV in the pre-HAART era [19], [20], little is known about the impact of pregnancy on response to HAART in Africa [21]. There are several biologically plausible mechanisms which might attenuate efficacy of several antiretroviral agents including changes in enzyme activity and beta-estradiol levels [22], [23], [24], [25], [26], pregnancy-related changes in blood volume and body mass, and factors which may compromise adherence to HAART ante- and post-partum, such as nausea/vomiting, labor-associated morbidity, or responsibility for a new infant.

We previously found that incident pregnancy is associated with increased risk of virologic failure in South Africa [11], but have been able to identify only a single study from Africa addressing the impact of pregnancy on mortality. This South Africa study found no association between pregnancy prevalent at the time of HAART initiation and subsequent mortality rate over three years [27]. However, as we have argued previously, using pregnancies prevalent at HAART initiation to make inference about causal effects of pregnancy may lead to misleading impressions due to selection bias [9], [28]. Here, we use a prospective cohort including over 800 incident pregnancies to assess the impact of incident pregnancy after HAART initiation on time to death or a joint outcome of a new AIDS diagnosis or death, as well as on time to becoming lost to follow-up (or drop-out).

## Methods

### Ethics Statement

This research based on de-identified secondary clinical data was approved by both the University of the Witwatersrand (Johannesburg, Gauteng, South Africa, Protocol M110140) and Duke University (Durham, North Carolina, USA, Protocol Pro00025267). The University of the Witwatersrand does not require written consent for retrospective reviews of de-identified data.

### Study Population and Design

We performed a retrospective analysis based on data from an observational cohort using the electronic patient database of the Themba Lethu Clinic. [29], [30] The Themba Lethu Clinic (henceforth, TLC) Cohort is a study of adults initiating HAART in Johannesburg, South Africa. The TLC sits within the regional Helen Joseph Hospital in urban Johannesburg, and is the largest single clinic providing HAART in South Africa, with over 18,000 patients currently in care (both receiving HAART and pre-HAART initiation). We study previously HAART-naïve women from the time of HAART initiation between 1 April 2004 and 31 March 2011 at TLC, and followed these women until they experienced end of care due to drop-out, death, or transfer of care to another site, or until administrative end of follow-up on 30 September 2011. Women were assumed to be HAART-experienced if they had a previously recorded antiretroviral therapy regimen, if they transferred in from another ARV-initiation site, if they were receiving second-line HAART (zidovudine, didanosine, and lopinavir-ritonavir) at baseline, or if they had a CD4 count >350 cells/mm^3^ or viral load ≤400 copies/ml at baseline. Additional exclusions (by age and baseline pregnancy status) are described below.

Typical first-line HAART included stavudine, lamivudine, and efavirenz; starting approximately 1 April 2010, stavudine was replaced with tenofovir in first-line HAART. Due to concerns about teratogenicity, women found to be pregnant in the first trimester are typically placed on the boosted protease inhibitor Kaletra (lopinavir and ritonavir) rather than efavirenz, while non-pregnant women with declared pregnancy intentions at baseline are placed on nevirapine rather than efavirenz. Additional details of the TLC clinical database, clinic procedures, and outcomes have been described previously [29], [31]; here we note only that clinical data are captured prospectively in the TLC and that accuracy of data entry has been previously validated. [29] In analysis, we “lagged” (delayed) changes in drug regimens by three months later to ensure that changes in drug regimen due to pregnancy were not mistaken for incident pregnancy due to a new drug regimen.

### Definitions and Data

The main exposure in this study was “ever became pregnant after HAART initiation” (hereafter, incident pregnancy); that is, pregnancy (completed or otherwise) which occurred subsequent to HAART initiation. As noted above, we have in previous work [11], [28] argued that apparent effects of prevalent pregnancy may be subject to selection bias [32], or alternately, confounding by indication. More specifically, women who start HAART during pregnancy are most often starting HAART because of pregnancy; these women are likely to be systematically different than women who started HAART for their own health and only later become pregnant. Thus, we excluded from analysis all women who were pregnant at baseline, and analyze only incident pregnancy as an exposure. Furthermore, since we are interested not only in the (approximately) 40 weeks of pregnancy but also in the long-term consequences of the experience of pregnancy, we define all person-time following the start of a first pregnancy during follow-up as exposed person-time. Incident pregnancies were identified from clinical records, as well as from records of antiretroviral drug regimens which list both pregnancy and “end of pregnancy” as reasons for regimen change.

The main outcome in this study was death; we considered a secondary outcome of death or a new AIDS defining event [33]. Deaths were obtained from the clinic database, and from the national death registry [34]. AIDS-defining conditions were obtained from clinical records; please see File S1 for details of this definition. Lost to follow-up was defined as not being seen in clinic for six months, no evidence of clinic transfer, and no record of death in the national death registry.

### Statistical Analysis

Baseline characteristics of women were described using simple statistics, including chi-square tests for categorical variables and Wilcoxon rank sum tests for continuous variables.

As noted above the main analysis focused on the effect of incident pregnancy on time to death. A key concern of this analysis was the possibility of time-varying confounding affected by prior exposure. [35], [36] This situation might manifest as follows: the effect of pregnancy on time to death is likely confounded by CD4 count (as a proxy for underlying immune status); but CD4 count might itself be affected by pregnancy. Situations in which time-varying confounding affected by prior exposure is likely are best analyzed using inverse probability weights to estimate marginal structural Cox proportional hazards models [35], [36] and confounding-adjusted extended Kaplan-Meier curves [37]. Inverse probability weights account for bias due to both confounding [38] and drop-out. [35] In the main analysis, we censored inverse probability weights at the 0.1^st^ and 99.9^th^ percentiles to reduce the overall variance of our estimates and to prevent single individuals who are exposed against expectations (e.g., a very immunosuppressed, older woman who becomes pregnant) to exert undue influence on results of analysis. [39].

In all multivariable analyses, we considered the following confounders of the effect of pregnancy on death (or AIDS and death), based on previous literature and plausible biological mechanism. Confounders measured at baseline (HAART initiation) included age, ethnicity, employment status, current tuberculosis, calendar date of HAART initiation, and WHO stage. Confounders measured over time included weight, body mass index, hemoglobin, CD4 count and CD4 percent, drug regimen, and drug adherence estimated from pharmacy visit data. We did not control for baseline or time-updated viral load because of the high proportion of missingness, but included a sensitivity analysis in which viral load was imputed. We used restricted four-knot cubic splines to flexibly control for age, body mass index, CD4 count, and time-on-study.

### Sensitivity Analysis and Missing Data

To test analytic assumptions, we performed three sensitivity analyses in addition to the main analysis; these sensitivity analyses addressed issues in definitions of the population, exposure, and outcome, as well as technical decisions in the modeling. The most critical sensitivity analyses were in exploring alternate outcome definitions. These analyses included 1) a combined outcome of death and new stage 4 clinical AIDS events and (separately) 2) combined death and new stage 3 or 4 clinical AIDS events [33]. Missing data led to approximately 18% missing observations in the final analysis, so we also conducted a multiple imputation analysis to account for missing baseline data [40]. In all analyses, longitudinal data were carried forward from the most recent observed value.

### Role of the Funding Source

The funding sources had no involvement in the design or conduct of the study, in the collection, management, analysis, or interpretation of the data, in the preparation, writing, review or approval of this manuscript, or in the decision to submit this manuscript for publication.

## Results

The initial study population comprised 7,534 non-pregnant, ART-naïve women ages 18–45, who contributed a total of 249,754 person-months, or 20,813 person-years of follow-up to this analysis, of which 2,472 (12%) person-years were exposed (occurring coincident with or subsequent to an incident pregnancy). Mean follow-up in all women was 2.7 years, and median (interquartile range) for follow-up was 2.1 (0.8, 4.3) years.

Baseline characteristics of the 7,534 women and characteristics of their contributed follow-up time are described in Table 1. The typical woman was 33 years old at initiation of HAART with a body mass index below 25 kg/m^2^ (and often below 18.5 kg/m^2^), low hemoglobin (median [IQR] 10.9 [9.5, 12.3] g/dL), and a CD4 count below 100 cells/mm^3^. Among the 19% of women who had a viral load taken at baseline, most (81%) had a viral load above 10,000 copies/ml. Over follow-up, most person-time was virally suppressed and at a CD4 counts above 200 cells/mm^3^.

**Table 1:** Characteristics of 7,534 women initiating HAART in Johannesburg, South Africa from 1 April 2004 to 31 March 2011, at study entry and contributed during 20,813 person-years of follow-up time.

Demographics	Subjects (n = 7,534 women)	Person-years (n = 20,813)
Follow-up time *years*	2.1 (0.8, 4.3)	
Baseline age *years*	33 (29, 38)	
Employed	3,259 (43.3)	
History of smoking	421 (5.6)	
Clinical		
Weight *kilograms*	57 (50, 66)	63 (55,73)
Body mass index *kg/m^2^*	22.2 (19.4, 25.7)	24.7 (21.8, 28.4)
Body mass index category *kg/m^2^*		
<18.5	1,248 (17.7)	1,233 (6.0)
18.5–24.9	3,774 (53.4)	9,479 (46.3)
25.0–29.9	1,366 (19.3)	6,051 (29.5)
≥30	682 (9.7)	3,721 (18.2)
WHO stage III or IV	2,816 (42.8)	
Current tuberculosis	1,254 (16.6)	
Drug regimen includes:		
Efavirenz	6,684 (88.2)	15,898 (76.4)
Nevirapine	655 (8.7)	1,790 (8.6)
Kaletra	246 (3.3)	2,904 (14.0)
Stavudine	6,093 (80.9)	12,228 (58.8)
Tenofovir	1,249 (16.6)	3,193 (15.3)
Laboratory		
Hemoglobin *grams/dl*	10.9 (9.5, 12.3)	12.4 (11.5, 13.6)
Hemoglobin, low^‡^ *grams/dl*	3,993 (55.9)	4,679 (22.8)
CD4 count *cells/mm^3^*	95 (36, 165)	317 (187, 473)
CD4 count category *cells/mm^3^*		
≤50	2,260 (31.9)	1,182 (5.8)
51–100	1,426 (20.1)	1,104 (5.4)
101–200	2,535 (35.8)	3,465 (16.9)
201–350	866 (12.2)	14,811 (72.0)
Viral load category^†^ *log copies/ml*		
≤400	(Excluded)	15,794 (88.1)
401–10,000	278 (19.2)	1,050 (5.9)
>10,000	1,167 (80.8)	1,086 (6.1)

Categorical variables are expressed as number (% total); continuous variables are expressed as median (interquartile range). P-values are two-sided by chi-square test, or Wilcoxon rank sum test. ^‡^ Number with hemoglobin below lower limit of normal (LLN); after adjustment for altitude, hemoglobin LLN is 11.35 (10.35) g/dl for non-pregnant (pregnant) women. ^†^ Baseline viral load was missing in 6,088 (81%) women as not standard of care.

**Figure 1:**
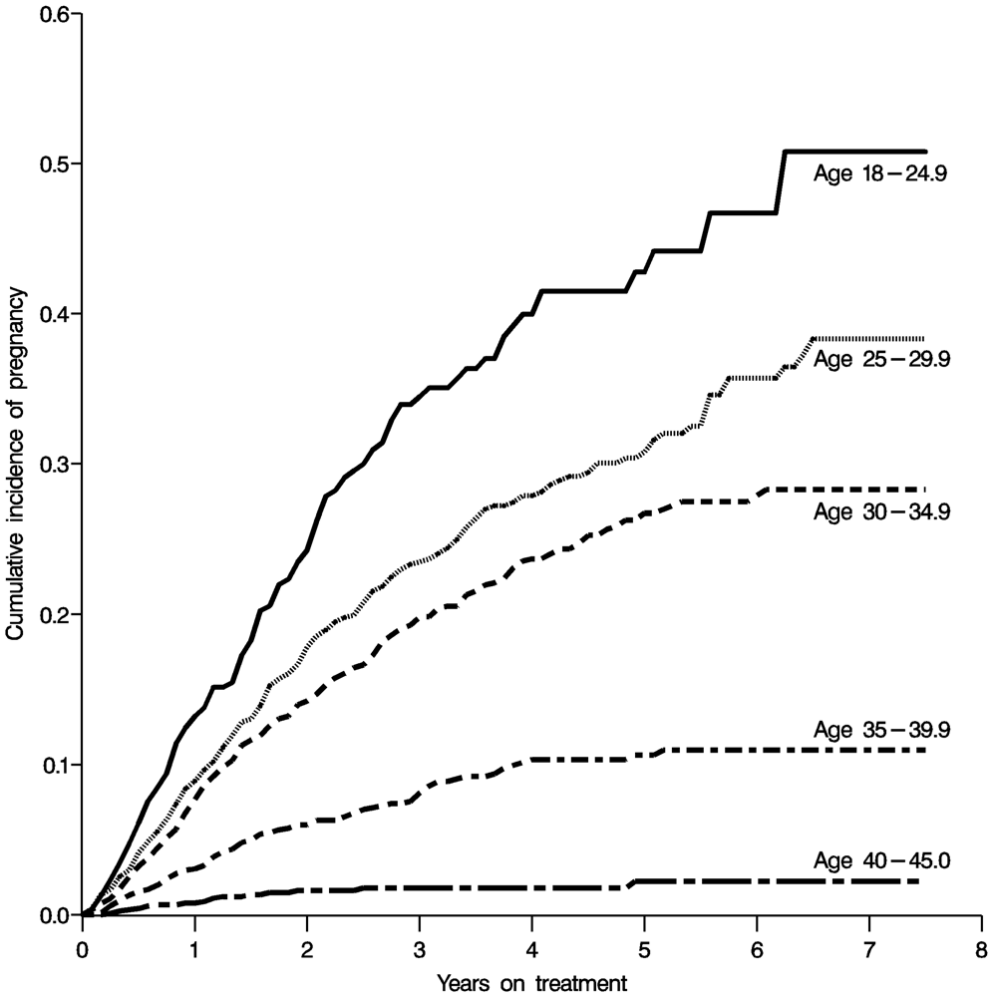
Cumulative risk of first incident pregnancy since HAART initiation, stratified by baseline age.

A total of 918 women (12%) experienced at least one pregnancy during follow-up, at a median of 14 (IQR 7, 26; mean 19) months after initiation of HAART. Younger women (18–25 years old) and those with higher CD4 counts were more likely to become pregnant during follow-up. Not surprisingly, women were more likely to become pregnant if they were receiving nevirapine or lopinavir-ritonavir compared with efavirenz. More detailed evaluation of factors contributing to incident pregnancy in this population can be found elsewhere [10]. Cumulative incidence of first pregnancy stratified by baseline age is shown in Figure 1. Of note, in women 25 years or younger at HAART initiation, over 50% have experienced at least one incident pregnancy by seven years of follow-up.

Observed adherence to HAART (estimated from pharmacy refill records) was high in all person-time: 89.2% of adherence assessments during non-pregnant person-time and 88.0% of adherence assessments during pregnant person-time showed 100% pill coverage (availability of an adequate drug supply, which is an upper limit on potential adherence) over the previous two months. Overall, we estimated that 95.0% of non-pregnant person-time and 94.4% of pregnant person-time was covered by an adequate drug supply.

Of 7,534 women considered in the main analysis, 21 women died after experiencing an incident pregnancy (2.3% of the 918 ever-pregnant women), and 614 died without experiencing incident pregnancy (9.3% of the 6,616 never-pregnant women). Among the 21 women who died after incident pregnancy, the median time between the start of the pregnancy and death was 15 months (IQR 8, 24), with only 8 women dying within nine months of the start of the pregnancy.

In the main analysis, the crude HR for the total effect of incident pregnancy on time to death over all of follow-up was 0.67 (95% CL 0.43, 1.05), and the weighted was 0.84 (95% CL 0.44, 1.60). Truncated inverse probability of treatment and censoring weights were well-behaved [39]. Results were similar (HR = 0.90, 95% CL 0.46, 1.78) when restricting to women alive and in care after six months. The crude analysis restricted to the population used in the weighted analysis (e.g., with no missing covariate data in the weights) gave HR = 0.56 (95% CL 0.33, 0.97), suggesting that any bias due to missing data was likely small in magnitude. Singly imputing time-updated log-viral load and controlling for this factor did not meaningfully alter the estimated effect. When limiting to patients with a valid recorded national ID (62% of participants), whose vital status should have been verifiable in the National Death registry, results were similar (HR = 0.79, 95% CL 0.39, 1.63).

Results were similar when expanding the outcome definition to include stage 4 clinical AIDS events, and (separately) stage 3 and 4 clinical AIDS events (Table 2). Results remained similar when restricting to those alive and in care at six months for those two alternate outcome definitions. Results were similar when restricting to women ages 18–35 (HR 0.87, 95% CL 0.44, 1.71). Multiple imputation analysis (n = 5 imputations) reduced proportion of missing data to 5.6%, and an overall result closer to the null of HR = 0.99 (95% CL 0.58, 1.68).

**Table 2:** Estimated effect of pregnancy on time to death and alternate outcomes among 7,534 women initiating HAART in South Africa, 2004–2011.

Death	No. of event‡	Person-months of follow-up	HR	95% CL
Unadjusted				
Not pregnant	614	220,093	1.	
Pregnant	21	29,661	0.67	0.43, 1.05
Weighted†				
Not pregnant	456	181,558	1.	
Pregnant	14	24,213	0.84	0.44, 1.60
**Death or AIDS Stage 4**				
Unadjusted				
Not pregnant	868	209,694	1.	
Pregnant	33	27,746	0.80	0.56, 1.15
Weighted†				
Not pregnant	677	172,832	1.	
Pregnant	21	22,759	0.87	0.51, 1.49
**Death or AIDS Stage 3/4**				
Unadjusted				
Not pregnant	1,105	199,123	1.	
Pregnant	40	25,603	0.92	0.67, 1.28
Weighted†				
Not pregnant	876	163,912	1.	
Pregnant	29	21,118	1.13	0.72, 1.78
**Lost to follow-up**				
Unadjusted				
Not pregnant	1,796	220,093	1.	
Pregnant	152	29,661	0.70	0.59, 0.83
Weighted†				
Not pregnant	1,349	181,558	1.	
Pregnant	121	24,213	0.62	0.51, 0.75

HR, hazard ratio; CL, confidence limit.

† Weighted models accounted for age, employment status, active tuberculosis at study entry, calendar date at entry, WHO stage, and baseline and time-updated measures of weight, body mass index, hemoglobin, CD4 count and percent, adherence, and current drug regimen.

‡ Difference from unadjusted model due to missing data in any variable; only complete observations get weights.

**Figure 2:**
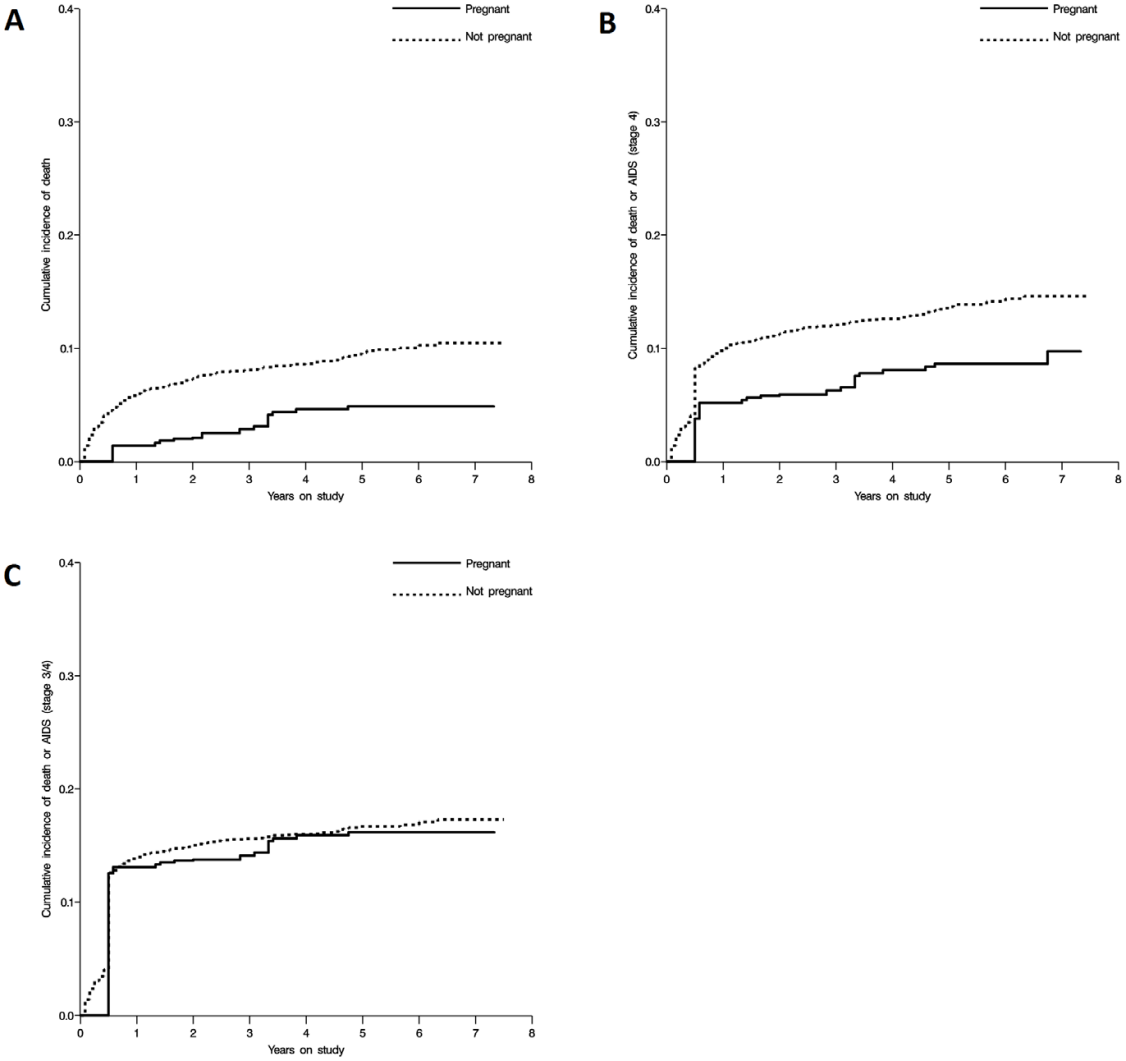
Effect of pregnancy on time to (A) death, (B) death or new stage 4 AIDS, or (C) death or new stage 3 or 4 AIDS. Curves are inverse, weighted, extended Kaplan-Meier curves.

Figure 2 shows the weighted extended Kaplan-Meier curves [37] for the effect of pregnancy on time to death (A), to death and clinical stage 4 AIDS events (B), and to death and clinical stage 3 or 4 AIDS events (C), all of which illustrate the findings from the Cox proportional hazards models.

**Figure 3:**
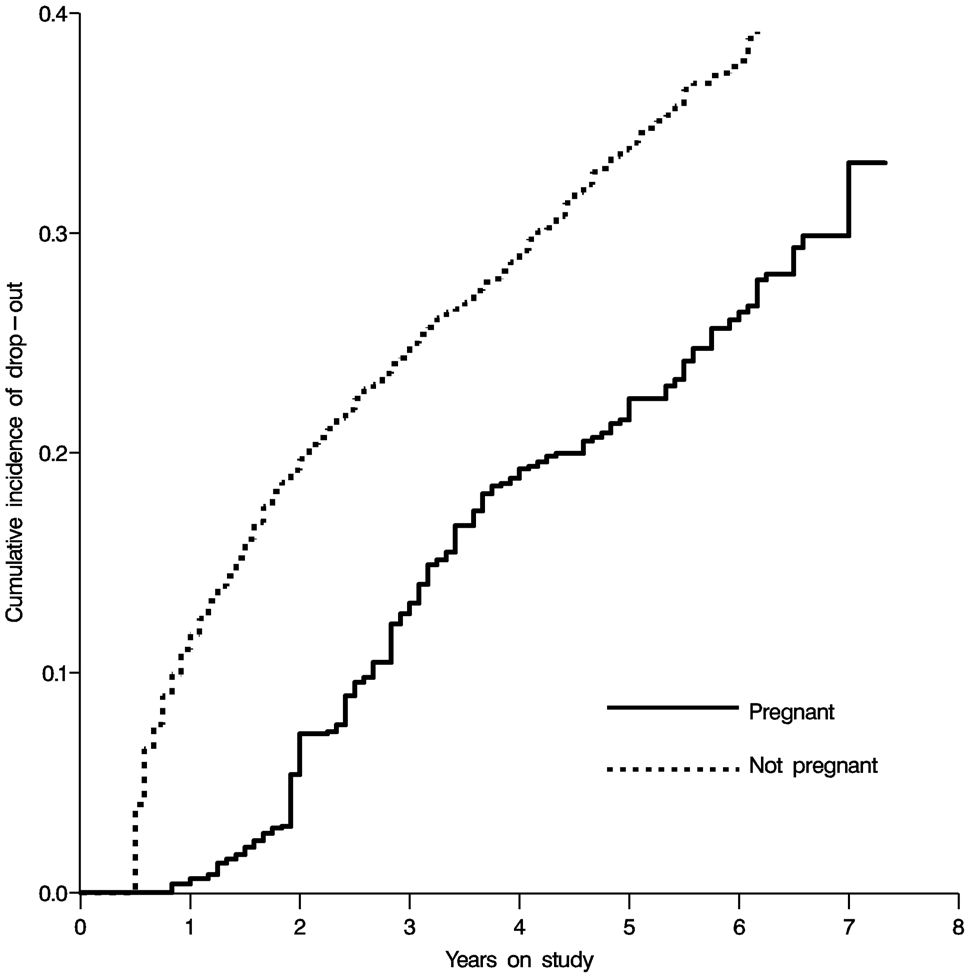
Effect of pregnancy on time to drop-out, displayed as weighted inverse extended Kaplan-Meier curves.

We also investigated the effect of incident pregnancy on drop-out (previous to death, not previous to new AIDS events), finding that pregnancy was associated with a reduced hazard of becoming lost to follow-up, HR = 0.62 (95% CL 0.51, 0.75). Figure 3 shows the weighted extended Kaplan-Meier curves for the effect of pregnancy on time to drop-out. Drop-out results were similar when restricting to person-time after the first six months.

## Discussion

In this observational study of HIV-positive women initiating HAART in South Africa, we found that pregnancy was not associated with an increased hazard and risk of death nor with increased hazard of combined outcome of AIDS or death over a substantial period of follow-up (Table 2). These results were generally supported by sensitivity analysis. While we estimated hazard ratios below or approximately at the null for the effect of pregnancy on death or AIDS, it seems unlikely that pregnancy is truly *protective* against death; rather, keeping in mind substantive considerations, we suspect that these results reflect a null effect of pregnancy on risk of clinical response to HAART, rather than a protective effect. Put another way, we believe that these results argue simply that pregnancy does not increase overall risk of death in this setting.

The clinical results largely cohere with reports from the United States [41] and South Africa [27], although both of those reports have some limitations. The US results are difficult to interpret due to methodological concerns [42], [43], while the South African study concentrated on prevalent pregnancy rather than incident pregnancy, an approach which may have significant limitations [32]. These results point in the opposite direction of previous results from this database showing that incident pregnancy may mildly accelerate rates of virologic failure [11], but are in line with another study of pregnancy and virologic failure [44]. As this suggests, much remains unknown about the impact of incident pregnancy on response to HAART (if any): more work is necessary to understand this relationship.

In contrast, we found a robust association of pregnancy and reduced hazard of drop-out from the cohort (HR = 0.62, 95% CL 0.51, 0.75). Unlike the clinical responses, the observed effect of pregnancy on drop-out may plausibly represent a protective effect of pregnancy. Such a protective effect might be observed if, for example, clinical providers were emphasizing the need to stay in care to new or expectant mothers, to protect the health of a newborn both directly (e.g., by preventing transmission) and indirectly (by preserving maternal health and thus enabling better care for the child). Of note, this finding stands in stark contrast to the association of prevalent pregnancy (at HAART initiation) with increased rates of lost-to-follow-up [27], [28]; however, some of that increased rate may be due to missed transfer rather than drop-out.

We saw little difference in crude drug adherence between non-pregnant and pregnant person-time, where we might expect to see higher adherence among pregnant women [45], [46], [47] (although not necessarily [48], [49]). Some of this difference may be due to the fact that prior studies generally separated the pregnant and postpartum periods (and saw differences between them) [50], whereas we did not separate these two periods (see Methods). As well, many existing studies have focused exclusively on prevalent, rather than incident, pregnancy: as prevalent and incident pregnancy appear dissimilar in their association with virologic failure rates [9], [28], we might likewise expect them to be dissimilar in their association with adherence. We note that a recent large meta-analysis found that only 74% of pregnant women (and still fewer postpartum women) had optimal adherence to HAART [50], underlining the urgent need to identify HIV-positive pregnant women at risk of low- or non-adherence [49], [50].

Beyond large sample size and a long follow-up period which included over 20,000 person-years of follow-up, there were several key strengths of this study. Data were collected prospectively in a previously validated clinic database. [29] Issues of time-varying confounding affected by prior treatment were dealt with appropriately [51], using marginal structural Cox proportional hazards models [35] and weighted Kaplan-Meier curves. [37] Outcome collection was aided by the availability of the national death registry [34], [52], and results were similar when restricting analysis to those patients who gave us a valid medical identification number (and thus whose vital status should have been captured in the registry). Finally, previous reports from this cohort [29], [53] suggest that results from the Themba Lethu Clinic are generally comparable to other large cohorts in sub-Saharan Africa; thus, we believe that the present results will have good generalizability to other urban HAART cohorts in sub-Saharan Africa.

The main limitation of this study was that, despite the large numbers of participants (7,534) and person-time (20,813 person-years), there were only very few exposed person-events (21 deaths in women who had experienced pregnancy), which limited power. However, one can also view this small number of exposed person-events as a result in itself: one which suggests that while it is not impossible that pregnancy during HAART is of clinical concern, it is unlikely to constitute a major overlooked public health issue in this context. One reason for these low numbers was that some number of pregnancies may have gone undetected due to miscarriage or early pregnancy loss. If such pregnancies were numerous, and strongly associated with both death, these results could be misleading: a more cautious interpretation of these findings, that *recognized* incident pregnancy was not associated with increased hazard of death or AIDS, may therefore be advised. That said, sensitivity analyses (not shown) suggest that unrecognized pregnancies would have to be very strongly associated with death (a much stronger association than was observed among recognized pregnancies) in order to substantively change our overall conclusions.

Other limitations of this study should be noted. We analyzed observational data from a clinical database, and thus uncontrolled confounding (in particular, by viral load, which was often missing and so was not controlled in main analysis) remains a possible threat to the validity of this study. Likewise, misclassification of exposure, outcome, and other factors cannot be ruled out. However, our sensitivity analyses support the results of our main analysis, and as noted above the TLC database has been previously validated for accuracy. [29] At the same time, we note that observational studies are often more generalizable than randomized controlled trials, which typically have stringent inclusion and exclusion criteria. Moreover, as an exposure pregnancy cannot be randomized either ethically or practically; thus, robustly analyzed longitudinal observational data is by necessity the gold standard for research that considers pregnancy as an exposure.

Finally, there were substantial missing data in the main analyses. However, sensitivity analyses, including multiple imputation of missing values, suggests strongly that our complete case analysis is not biased in such a way as to alter the overall qualitative conclusions of this work. That said, we are missing information on precise causes of death, as well as on birth complications: efforts are ongoing to obtain the latter data.

In this large study of clinical cohort data, we found that recognized pregnancy after HAART initiation was not associated with increased relative hazard or absolute risks of death or AIDS, and may have decreased risk of drop-out. Nonetheless, this study reconfirms that incident pregnancy is common after initiation of HAART among younger women [8], [10], and emphasizes the need for better integration of reproductive healthcare services and standard clinical care for HIV-positive women in sub-Saharan Africa.

## Supporting Information

File S1 Defining New Clinical AIDS Events.
(DOCX)
