# Supplementary material for: Incident Pregnancy and Time to Death or AIDS among HIV-Positive Women Receiving Antiretroviral Therapy
Source: PLoS One. 2013 Mar 8;8(3):e58117. doi: 10.1371/journal.pone.0058117 (PMC3592862; doi:10.1371/journal.pone.0058117)
Supplement: File S1 — Defining New Clinical AIDS Events. (DOCX) [file pone.0058117.s001.docx]

**SUPPORTING INFORMATION S1: Defining New Clinical AIDS Events**

AIDS defining conditions were defined as in the WHO document “WHO Case Definitions of HIV for Surveillance and Revised Clinical Staging and Immunological Classification of HIV-Related Disease in Adults and Children” [[33](#_ENREF_33)], and classified as either WHO stage 3 or stage 4 events as in Table 3 in that document [[33](#_ENREF_33)] (titled, “WHO clinical staging of HIV/AIDS for adults and adolescents with confirmed HIV infection”). To these criteria we added “immunologic failure” as a stage 3 condition, defined as a CD4 count< 200 cells/mm^3^ after achieving a CD4 count ≥200 cells/mm^3^.

Because many women entered care with extreme immunosuppression and wasting, we were concerned that early AIDS events (e.g., a diagnosis of tuberculosis at month 2) might in fact be revealed, rather than new, AIDS conditions – that is, conditions the patient had when she entered care, not a new event. To prevent this, any clinical AIDS event (e.g., new diagnosis of extrapulmonary tuberculosis, Cryptococcal meningitis, etc.) that occurred in the first three months of follow-up was excluded as an endpoint. Laboratory AIDS events (neutropenia, thrombocytopenia, anemia) likewise had an exclusion period of three months. Finally, assessing severe weight loss (and therefore wasting) and immunologic failure was impossible without a baseline weight; we set baselines at six months post-baseline. Thus, a loss of 10% from six month weight anytime after month six was considered “severe weight loss” (clinical stage 3).
